# From Testers to Cocreators—the Value of and Approaches to Successful Patient Engagement in the Development of eHealth Solutions: Qualitative Expert Interview Study

**DOI:** 10.2196/41481

**Published:** 2022-10-06

**Authors:** Christine Jacob, Steven Bourke, Sabina Heuss

**Affiliations:** 1 FHNW - University of Applied Sciences Northwestern Switzerland Windisch Switzerland; 2 PersonalPulse GmbH Basel Switzerland; 3 FHNW - University of Applied Sciences Northwestern Switzerland Olten Switzerland

**Keywords:** telemedicine, smartphone, mobile phone, electronic health record, public health practice, technology, perception, health education, mobile health, mHealth, telehealth, eHealth, patients, patient engagement, patient voice, patient empowerment

## Abstract

**Background:**

Research has shown that patient engagement is most commonly done at the beginning of research or to test readily available prototypes and less commonly done in other phases such as the execution phases. Previous studies have reported that patients are usually assigned a consultative rather than a decision-making role in health service planning and evaluation.

**Objective:**

This study had 2 objectives: to better understand the challenges and opportunities in the inclusion of patients in the development of eHealth technologies and ideas on how to overcome the identified gaps and to create a research-based end-to-end practical blueprint that can guide the relevant stakeholders to successfully engage patients as cocreators in all human-centered design phases rather than mere testers of preplanned prototypes.

**Methods:**

Key informant interviews were conducted using in-depth semistructured interviews with 20 participants from 6 countries across Europe. This was followed by a focus group to validate the initial findings. Participants encompassed all the relevant stakeholder groups including patient experts, eHealth experts, health technology providers, clinicians, pharma executives, and health insurance experts.

**Results:**

This study shows that engaging patients in eHealth development can help provide different types of value; namely, identifying unmet needs, better usability and desirability, better fit into the patient journey, better adoption and stickiness, better health outcomes, advocacy and trust, a sense of purpose, and better health equity and access. However, the participants agreed that patients are usually engaged too late in the development process, mostly assuming a sounding role in testing a ready-made prototype. The justification for these gaps in engagement is driven by some prominent barriers, notably compliance risks, patient-related factors, power dynamics, patient engagement as lip service, poor value perception, lack of resources, mistrust, and inflexibility. On the positive side, the participants also reflected on facilitators for better patient engagement; for instance, engaging through engagement partners, novel approaches such as the rise of professional patient experts, embedding patients in development teams, expectation management, and professional moderation services.

**Conclusions:**

Overcoming the current gaps in patient engagement in eHealth development requires consolidated efforts from all stakeholders in a complex health care ecosystem. The shift toward more patient-driven eHealth development requires education and awareness; frameworks to monitor and evaluate the value of patient engagement; regulatory clarity and simplification; platforms to facilitate patient access and identification; patient incentivization, transparency, and trust; and a mindset shift toward value-based health care.

## Introduction

### Background

There is growing evidence that patients who are better informed and more engaged in their own care are more likely to be knowledgeable, stick to their treatment plans, and have a better quality of life [1]. Contributing to the research about patient engagement in health innovation, such as eHealth tools, supports the paradigm shift needed to normalize the patient’s role beyond “subject” or “participant” to “partner and cocreator” to the development of more effective eHealth solutions and ultimately lead to better health outcomes [2]. The insights provided by the individuals who care for and live with the disease on a daily basis are invaluable when it comes to creating innovative solutions that will be accepted and address unmet needs. However, patient engagement is often considered complex [3], and the definition of the term is not always clear, implying that different stakeholders may have a different understanding and different expectations of what patient engagement is [4]. This study adopts a definition that encompasses the key attributes of patient engagement, “personalization, access, commitment, and therapeutic alliance,” and defines it as “the desire and capability to actively choose to participate in care in a way uniquely appropriate to the individual, in cooperation with a health care provider or institution, for the purposes of maximizing outcomes or improving experiences of care” [5].

Given its intricacies, research has shown that patient engagement is most commonly done at the beginning of research or to test readily available prototypes and less commonly done in other phases such as the execution phases [6]. Previous studies report that patients usually assume a consultative rather than decision-making role in health service planning and evaluation [7]. Nonetheless, true patient empowerment necessitates a shift from a *patients as testers* mentality to *patients as equal partners and cocreators*, which can be achieved by involving them in every step of the human-centered design (HCD) process. This may help optimize resources [8] and facilitate the development of health tools that address patients’ real unmet needs [9], using their experience and knowledge of their condition and not solely based on professionals’ perceptions of patients’ needs.

Different barriers, including a lack of insight into appropriate engagement methods, may be limiting patient involvement in eHealth innovations development activities [10]. More research is required to validate the expressed views among the different stakeholders in the health care ecosystem to establish effective methods for engaging patients [7]. This work builds on the efforts made in traditional clinical research, such as the road map developed by Geissler et al [11], with the aim of extending them to eHealth through a HCD approach, which is still in its infancy when it comes to systematic patient engagement in health care innovations [12-14].

Engaging users in the development of eHealth tools is a critical factor for their success, as it safeguards their usability and safety [15]; this is reflected in how institutions such as the Food and Drug Administration demand evidence of end-user engagement in health technology design when reviewing market presubmissions [16]. There is a greater need to assist patients in the daily management of their disease, for example, by helping them develop better adherence to treatments, resulting in better care outcomes. Digital tools strive to provide opportunities for this management; however, current findings demonstrate that most mobile health apps do not necessarily increase medication adherence [17]. Therefore, in recent years, there has been a growing body of research involving users in the development of health care technologies in what is called HCD.

The term “user-centered design” was first coined by Donald Norman in the 1980s to refer to a design philosophy that puts technology users at the center of the development process [18]. In 2010, the ISO 9241-210 extended the definition to also include other stakeholders beyond the direct users of the technology and referred to this new approach as HCD. The standard describes the key benefits of the HCD approach by explaining that “usable systems can provide a number of benefits, including improved productivity, enhanced user well-being, avoidance of stress, increased accessibility, and reduced risk of harm” [19]. These concepts are closely related to universal design, which aims to develop accessible technologies for all users regardless of their physical or cognitive capabilities [20], creating an inclusive design that takes into account the often overlooked patient populations that may be facing physical or cognitive challenges due to their health conditions.

In health care studies, the user-centered design ISO 9241-210 for HCD of interactive systems [19], the HCD IDEO Field Guide to Human-Centered Design [21], and the Hasso Plattner Institute School of Design Thinking [22] are among the most used frameworks for this purpose [13]. These frameworks have some overlaps, and when aggregated, they would cover five key phases: (1) specifying the context and evidence review, (2) defining user requirements and user research, (3) producing the design and testing the concept, (4) prototyping and testing against the initial requirements, and (5) delivering the solutions and usability testing. However, eHealth providers and developers are often faced with factors such as time pressure and rapid development life cycles that render the structured and iterative nature of HCD challenging to apply in practice [15].

### Objectives

This study had 2 objectives: to better understand the challenges and opportunities in the inclusion of patients in the development of eHealth technologies and ideas on how to overcome the identified gaps and to create a research-based end-to-end practical blueprint that can guide the relevant stakeholders to successfully engage patients as cocreators in all HCD phases rather than mere testers of preplanned prototypes. The resulting blueprint aims to support the key stakeholders across the health care ecosystem to systematically cocreate with patients and assist in developing eHealth solutions tailored for people in specific contexts and with specific needs. This allows for ethical designs that respect privacy and quality of life and reduce the chances of situations with a high risk of human error, leading to the creation of more relevant and safer tools that are more likely to be adopted by their intended users for better health outcomes.

## Methods

### Overview

In this study, we adopted a qualitative paradigm, which has become more common in research concerned with the assessment of health technologies as well as health services; this was reflected in the rising numbers of qualitative research published in medical journals [23]. One of the reasons behind the growing importance of qualitative methods in health care research is that they enable us to understand the complexities of today’s health care ecosystem by touching on complex social aspects such as user attitudes and behaviors in ways that cannot be reached by quantitative methods [24].

### Scope and Conceptual Framework

The World Health Organization defines eHealth as “the cost-effective and secure use of information and communications technologies in support of health and health-related fields, including health care services, health surveillance, health literature, and health education, knowledge and research” [25]. This study focuses on patient-facing eHealth tools, including self-management tools and remote eHealth solutions, and excludes tools with no patient interface, such as those used within and between care providers (eg, health care provider videoconferences or electronic health record integration), or health data analytics systems used at the population level.

Human-centric design has been chosen as the conceptual framework because it places the people we are trying to serve at the center and offers them the space to become partners in eHealth innovation. It is an iterative approach in which the focus is on understanding the dynamics between stakeholders across the ecosystem and cocreating with them. The framework allows for a systematic investigation of the gaps and possible engagement opportunities for each step of the design process, rather than only the testing phase, as is commonly the case. This systematic approach enables a better understanding of the barriers to patient engagement in the phases where they are currently least involved and discusses opportunities for better engagement strategies that cover all design phases.

### Sampling Strategy and Participant Recruitment

As in most qualitative studies, this research used purposive sampling with the objective of generating rich insights [26]. Potential participants were recruited based on their ability to provide rich and in-depth information about the research topic; they had to be individuals who have personal experience with the topic being studied so that they can articulate their real-life experiences [26,27]. The main selection criteria were that participants must belong to one of the key relevant stakeholder groups (ie, patient experts, patient organizations, eHealth providers and developers, clinicians, pharmaceutical experts, payers, and health tech researchers) and must have eHealth knowledge and experience to ensure a comprehensive view that takes the different perspectives into account when identifying the existing gaps and challenges and how to overcome the existing gaps and challenges with strategic patient engagement points and their realization to extend the benefit for all involved parties.

After shortlisting the participants of interest, as per the criteria explained earlier, the researchers contacted the key informants. To minimize potential selection bias, the researchers worked with the key informants to identify suitable participants in their network, a sampling technique called snowballing, where the researcher builds the sample through the network of other participants, in this case, the key informants [26]. As for the sample size, it is common in qualitative research aiming to identify patterns throughout data to recruit a sample somewhere between 15 and 30 interviews [26]; therefore, the researchers aimed to recruit enough participants, with the aim of reaching saturation, which is usually a signal that enough data have been collected, which is when new data do not generate new insights anymore [26,28-30].

Table 1 presents the demographics and characteristics of the sample. Several participants had multiple backgrounds; for example, some were pharmaceutical executives who worked in pharmaceutical companies and then moved to work for a big tech company. They combined both backgrounds and shared insights that capitalized on their experiences in both worlds. Participants categorized as eHealth experts have combined expertise in developing, conceptualizing, or testing eHealth tools. The participant, categorized as a health insurance expert, works for a health insurance company and has in-depth expertise in eHealth assessment and reimbursement criteria. Those categorized as health care professionals are participants with a clinical background. The participant categorized as a patient advocate is not a patient but rather an expert involved in patient organizations and actively working on patient engagement initiatives, such as assessment frameworks. Patient experts combine their disease knowledge and experience with eHealth relevant professional expertise and skills such as software development and user experience. Pharmaceutical executives are participants who work or have previously worked for a pharmaceutical company. Furthermore, those categorized as technology providers are participants who work in either an eHealth startup or a big tech company that focuses on eHealth.

**Table 1:** Sample demographics and characteristics (N=20).

Demographics and characteristics			Values, n (%)
**Background (some participants had multiple backgrounds)**			
	eHealth experts	6 (30)	
	Health insurance experts	1 (5)	
	Health care professionals (clinicians)	3 (15)	
	Patient advocates	1 (5)	
	Patient experts	7 (35)	
	Pharmaceutical executives	5 (25)	
	Technology providers (big tech and startups)	7 (35)	
**Sex**			
	Female	11 (55)	
	Male	9 (45)	
**Location**			
	Belgium	1 (5)	
	Germany	2 (10)	
	Ireland	4 (20)	
	Italy	1 (5)	
	Switzerland	9 (45)	
	United Kingdom	3 (15)	

### Data Collection and Synthesis

Data were collected via in-depth, semistructured interviews conducted on the web. Data collection took place from March to May 2022, and a total of 20 participants located in 6 countries across Europe (Switzerland, Germany, the United Kingdom, Ireland, Belgium, and Italy) were interviewed. The median interview duration was 62 minutes, resulting in 291 pages of transcribed interview data for the 20 interviews.

The high-level research questions that helped guide the one-on-one interviews with the relevant stakeholders to create the blueprint are as follows:

- What is the value of engaging patients in the development of eHealth technologies?
- What are the barriers to and facilitators of patients’ engagement in eHealth tools development?
- What are the gaps in the current patient engagement approaches (ie, the development phases in which they are least involved)? What can be novel approaches to patient involvement in cocreation to overcome the current gaps?

These research questions resulted in an interview guide composed of 12 questions and a maturity assessment survey that reflected the 5 stages of the human-centered approach to design. A copy of the interview guide is included in Multimedia Appendix 1, and a copy of the Survey Monkey form for maturity assessment is included in Multimedia Appendix 2.

Data coding began with a preliminary data extraction grid that included themes informed by previous research, the systematic steps in the HCD framework, and the research team’s previous work on the topic. More themes were added as they emerged during the data analysis process. The thematic analysis by Braun and Clarke [28,29] was used to identify and extract themes addressed in the research questions. Computer-assisted qualitative data analysis software, Atlas.ti, was used for data coding. The first author (CJ) conducted the interviews and performed the initial analysis and coding. The second author (SB) reviewed the coding, and any cases of disagreement were discussed in conjunction with the last author (SH) and mutually agreed upon. The phases of thematic analysis are explained in detail in Multimedia Appendix 3. This process lasted from May to July 2022.

After generating the initial results, the researchers shared and discussed them in a web-based focus group with the same participants to ensure the validity and reliability of the findings and capture any potential additional insights that the participants may add [31]. The focus group was recorded and analyzed using the same method used for the in-depth interviews.

### Role of the Researchers

Researchers play a fundamental role in qualitative research; this is because they are considered the instrument of the research, and accordingly, the analysis and findings are impacted by their approach and the way of evaluating and understanding things [26,31]. This does not mean that anything would be accepted in qualitative research, as some critiques say, but rather that the researchers “tell one story among many” that could be told about these specific data [26].

The practice partner, (SB), the founder of PersonalPulse, is delivering transformation in citizen-led health care innovation, working together with relevant stakeholders in the health care ecosystem to cocreate health care solutions that are relevant, usable, and sustainable [32]. PersonalPulse is run by patients, is run for patients, and collaborates with a wide network of patient experts in diverse disease areas to give them a voice and empower them as equal partners in the creation of new health care solutions. The research partners (CJ and SH), from the University of Applied Sciences, Northwestern Switzerland, are both seasoned health care experts and researchers, with vast health care experience in hospitals, pharmaceutical companies, and health care technologies both from practice and research perspectives.

This background empowered the research team with a strong and wide network in health care and enabled them to access key informants in the area of eHealth. This helped them with access to participants and also fostered a relaxed and mutually beneficial dialogue between them and the key informants. To minimize the risk of researcher bias during the interviews, the interviewers refrained from stating their own views on the matters being discussed to minimize the likelihood of a directive discussion and to enable the participants to freely express their opinions [33].

### Ethical Considerations

The Ethics Committee of Northwest and Central Switzerland determined that ethics approval was not needed for this study, according to the Federal Act on Research involving Human Beings, article 2, paragraph 1 (reference number Req-2022-00119). All participants were briefed about the research background and signed a consent form agreeing to participate.

## Results

### The Meaning and Value of Patient Engagement in the Development of eHealth Technologies

**Figure 1:**
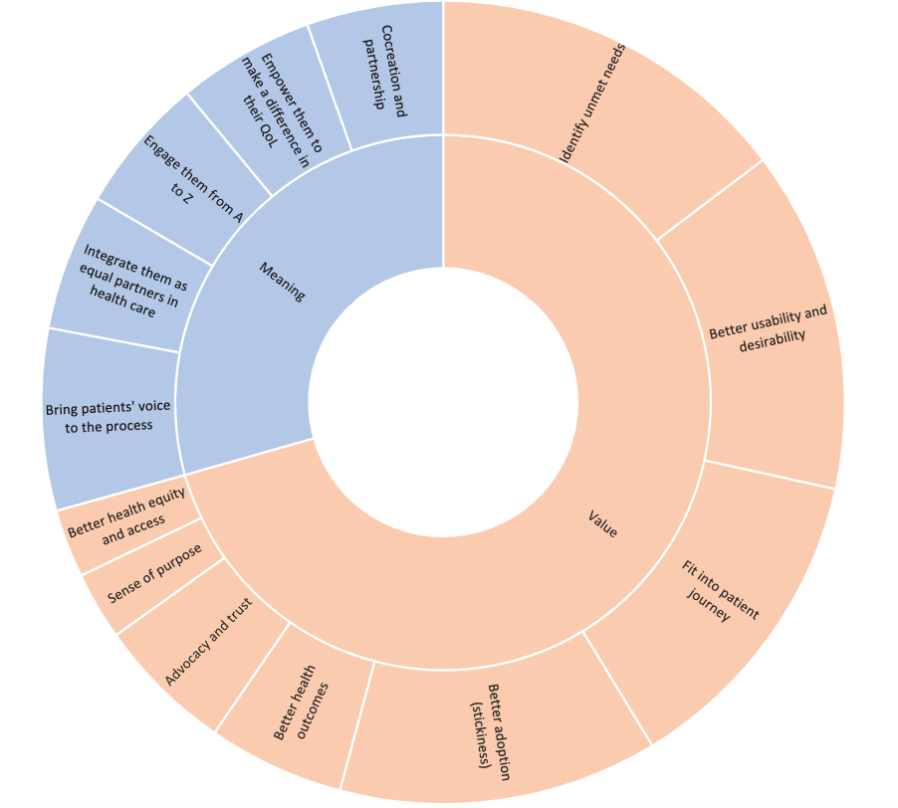
The meaning and value of patient engagement in eHealth development. QoL: quality of life.

As a first step, we wanted to better understand how the expert participants define good patient engagement and the different types of value that it may generate. Figure 1 shows the themes that emerged as a response to these 2 key questions and their respective subthemes, reflecting the frequency of each theme (frequencies reflect the number of participants who mentioned that specific theme).

When asked about their definition of good patient engagement in eHealth development, many participants said that in its simplest form, it is about bringing patients’ voices to the process (8/20, 40%), but most of them went a step further to explain that it is also about real cocreation and partnership (6/20, 30%), empowering patients to make a difference in their quality of life as a whole (6/20, 30%), engaging them in the whole process from beginning to end (6/20, 30%), and integrating them as equal partners in the development process (6/20, 30%).

Truly engaging patients as equal partners in eHealth development can bring different types of value. Most participants agreed that one of the most prominent values it can bring is the ability to identify unmet needs (16/20, 80%), followed by better usability and desirability of the tools (15/20, 75%), which resulted in better adoption and stickiness (14/20, 70%), and a more holistic view that enables a better fit into the overall patient journey (14/20, 70%). It also fosters trust and advocacy (6/20, 30%). The tools’ better adoption and stickiness also imply better health outcomes because of adherence (6/20, 30%), which enables the least technically capable patients to still be able to use those tools, resulting in better health equity and access (3/20, 15%). It also gives a sense of purpose to the developing team as they can relate better to the patients’ needs and pain points (3/20, 15%).

These key themes and subthemes, their frequencies, and sample quotes about the meaning and value of patient engagement are summarized in Table 2.

**Table 2:** The meaning and value of patient engagement as expressed by the participants (N=20).

Theme			Sample quotes
**The meaning of patient engagement**			
	Bring patients’ voice to the process (n=8, 40%)	“A much greater focus on getting the patient voice and really not even just the patient voice, but the diversity of the input and running everything by getting everything prototyped in a design way before we go into writing a line of code” [P13-HCP-TP] “...you just need to have space and allow them to be in such a workshop and hear their voice and listen to them describing that” [P4-TP-Ph]	
	Cocreation and partnership (n=6, 30%)	“Ideally, you co-develop things with them, not just you just get their perspective, and that’s it because it’s also at the same time, you’re also changing their behavior” [P16-HCP-Ph] “I think it’s really about partnering, right? We’re partnering at eye level with another expert, let’s say, in what this person has gone through or is experiencing in their daily life” [P9-Ph]	
	Empower them to make a difference in their QoL^a^ (n=6, 30%)	“I think their involvement is always crucial because that should always be the overall objective, really improving patient life” [P12-DE] “...really to empower patients and enable patients to look after their own health” [P13-HCP-TP]	
	Engage them from A to Z (n=6, 30%)	“For me, patient engagement is, I think, about involving patients of every type at every level, at every point” [P15-TP] “I don’t see patient engagement as—I do my project and now I send you a questionnaire. And now I ask you, do you like it?—That’s for me, not patient engagement. Patient engagement is having them all the way.” [P19-PE-DE]	
	Integrate them as equal partners in health care (n=6, 30%)	“Good patient engagement, it’s putting the patient at the—give them a seat on the table at the same level as everyone else” [P19-PE-DE] “I think about patient engagement, I consider it as the same level interaction between patient and the provider environment” [P20-PE-DE]	
**The value of patient engagement**			
	Identify unmet needs (n=16, 80%)	“So it just brings the value of not just understanding what is needed, but understanding how it’s needed, and when it’s needed, and what could actually be used afterwards” [P19-PE-DE] “you get really a feeling of is this really something the patient would use afterwards, or need in their life, in their daily living, and in their world” [P5-PE-DE]	
	Better usability and desirability (n=15, 75%)	“The objective is to have the best solution, the most usable and effective solution.” [P10-DE] “If you can basically involve patients with different types of levels of understanding earlier on in the process, you’re more likely to get a product that actually is tailored to everybody’s familiarity.” [P11-PE]	
	Better adoption (stickiness; n=14, 70%)	“Ideally, you co-develop things with them, not just you just get their perspective, and that’s it because it’s also at the same time, you’re also changing their behavior...it’s more sustainable” [P16-HCP-Ph] “The most important thing is that you are sure that there’s an acceptance of what you’re doing, that patients, in the end of the day, take your product, your concept, your whatever because they want to use it” [P18-HCP-DE]	
	Fit into patient journey (n=14, 70%)	“In order to build solutions that solve real world problems for patients, then you need to use this really deep insight into the person behind the disease and in the context of their daily life. And honestly you can’t do that without working very closely with the patient” [P6-PE] “We need a patient journey that is way, way, way more easy than it used to be, way more rewarding, more nudging... So where can patients help in patient engagement? I think they really need to be there to describe the reality” [P8-TP-Ph]	
	Advocacy and trust (n=6, 30%)	“If you have contributed to developing and you see that this has been developed by also patients like you, then you are more also prone to use it” [P2-PE-DE] “This will help with adoption in the end, because now, yeah, we’ve created ambassadors, right? We don’t only work on this project with the community, but still, everybody owns this now. All the co-creators own this solution and they’re just waiting to share it with everyone” [P9-Ph]	
	Better health outcomes (n=6, 30%)	“Patient engagement is being able to sustain that change in behavior over time to get the clinical outcomes” [P13-HCP-TP] “So I guess in terms of impact as well on people’s life and really improving your health or at least the daily life and managing symptoms” [P2-PE-DE]	
	Better health equity and access (n=3, 15%)	“They’re able to augment the face to face with the digital platform, they’re able to use it as an extender of care” [P13-HCP-TP]	
	Sense of purpose (n=3, 15%)	“Having that engagement creates a more powerful purpose for the team” [P14-TP-Ph] “The people developing or thinking of building a solution, if they feel somehow identified with the patient, there’s an energy in the team which you have not seen before...it’s really identifying with the goal to find solution for this problem” [P1-In]	

^a^QoL: quality of life.

### Barriers and Facilitators for Patient Engagement in the Development of eHealth Technologies

**Figure 2:**
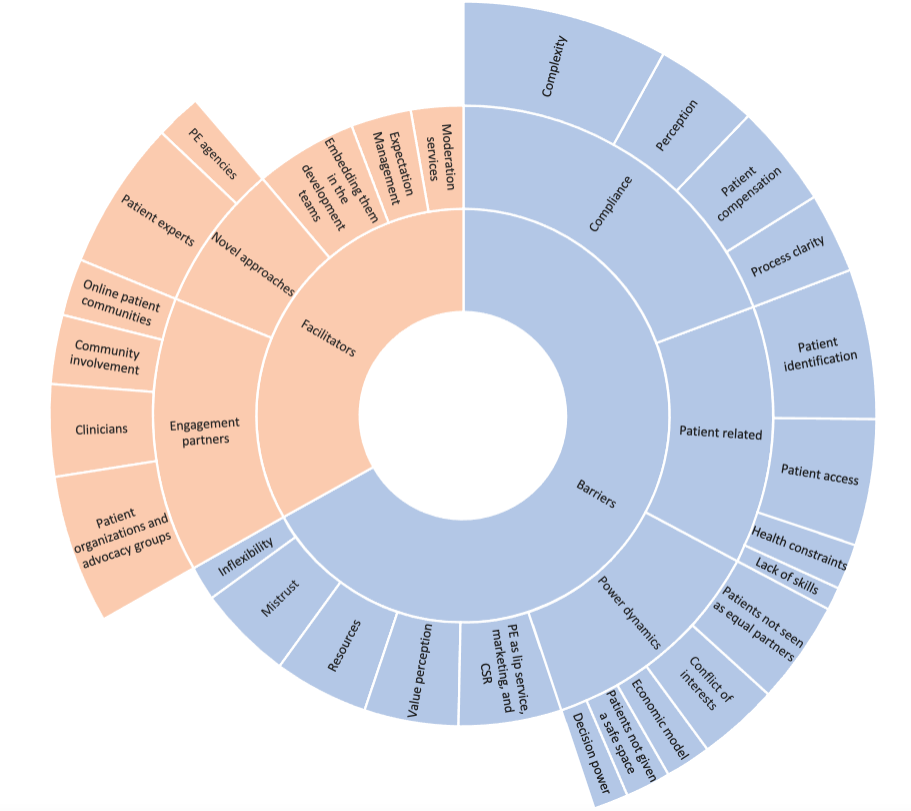
Barriers to and facilitators of patient engagement in eHealth development. CSR: corporate social responsibility; PE: patient engagement.

We then discussed the participants’ experiences with the most prominent barriers to and facilitators of engaging patients in eHealth development. Figure 2 shows the themes that emerged as a response to these 2 questions and their respective subthemes, reflecting the frequency of each theme (frequencies reflect the number of participants who mentioned that specific theme).

Barriers to patient engagement in eHealth development revolved around 8 key themes: compliance and regulatory, patient-related factors, power dynamics in the health care sector, patient engagement as lip service or corporate social responsibility, value perception, resources, mistrust, and lack of flexibility.

Compliance was the most prominent barrier, with participants mentioning the complexity of regulatory processes as a key hurdle (18/20, 90%). However, some participants pointed out that this may also be partly a perception issue in that some stakeholders may perceive compliance as more complex than it really is (9/20, 45%). Compliantly compensating patients for their engagement was also perceived as a hindrance (9/20, 45%), and lack of process clarity was also raised as an issue, especially for smaller eHealth providers that may not have the resources or in-house knowledge about all regulatory processes (7/20, 35%).

Participants expressed that some patient-related factors may also make it difficult to engage patients in eHealth development. Specifically, not only are patient identification (13/20, 65%) and patient access (11/20, 55%) a key hurdle but also some health-related constraints may render it difficult for some patients to engage (4/20, 20%) or some patients’ lack of the needed skills to engage efficiently (1/20, 5%).

Power dynamics in the health care sector may also hinder patient engagement. Patients not being seen as equal partners (9/20, 45%), conflict of interests among the stakeholders (7/20, 35%), the economic model (4/20, 20%), patients not being given a safe space to express their needs and pain points (4/20, 20%), and the lack of decision power in many cases (3/20, 15%) were the most prominent subthemes mentioned by the participants in this regard.

Other barriers included patient engagement being considered a marketing activity or lip service by some of the stakeholders (12/20, 60%), the lack of clarity on the value that patient engagement may bring (11/20, 55%), resource constraints (11/20, 55%), mistrust between patients and some other stakeholders (11/20, 55%), and sometimes a mere inflexibility of some eHealth providers (4/20, 20%).

These key themes and subthemes, their frequencies, and some sample quotes about barriers to patient engagement are summarized in Multimedia Appendix 4.

When asked about facilitators of patient engagement in eHealth development, most participants talked about working with different types of engagement partners to overcome some of the barriers such as compliance and patient identification and access. Patient organizations and advocacy groups were the most cited engagement partners (13/20, 65%), followed by clinicians (8/20, 40%), involvement in the patient community in general (6/20, 30%), and web-based patient communities (5/20, 25%).

Many participants also mentioned the rise of some novel approaches that play an active role in facilitating patient engagement, such as professional patient experts (13/20, 65%) and patient engagement agencies that play the role of matchmaking patients with the relevant stakeholders interested in engaging them (4/20, 20%).

Other approaches that may enable patient engagement include embedding them in the development team (12/20, 60%), managing patient expectations from the beginning to avoid disappointment in case some of their requests were not feasible or not in scope (7/20, 35%), and using professional moderation services that can help translate technical language to the patients and also help technical staff to understand the real needs of patients (6/20, 30%).

The key themes and subthemes, their frequencies, and sample quotes about the facilitators of patient engagement are summarized in Table 3 for clarity.

**Table 3:** Facilitators to patient engagement as expressed by the participants (N=20).

Theme			Sample quotes
**Engagement partners**			
	Patient organizations and advocacy groups (n=13, 65%)	“I also think that patient advocacy groups, of course, can support a lot because they can act as an important connector.” [P12-DE] “there’s lots of good societies and charities that work with patients, you could approach them directly, try get involved with them.” [P3-TP]	
	Clinicians (n=8, 40%)	“The key thing there is it was the clinicians that had this trusted relationship with the patients and ask them to do this.” [P13-HCP-TP] “So, to find a patient through a specialist who knew which patient he could ask for and also knew how to bring them into a group and introduce them.” [P1-In]	
	Community involvement (n=6, 30%)	“I think being more involved in the community that you’re serving as well. So as a company, taking part in charity events relating to the health area, maybe, that you’re working in. Going along and volunteering at events, opening up opportunities.” [P15-TP] “And even being present in forums... how are we present in those discussions? Patients will find themselves in communities, social media, all of that. And that’s a great way of scoping out new opportunities also. But somehow the organizations need to be present in those discussions.” [P4-TP-Ph]	
	Web-based patient communities (n=5, 25%)	“So, I think partnership with the patients, so whether it’s a patient organization or the online patient community, but for me, I always say I think it needs to be a mixture because it needs to truly represent the community as a whole.” [P16-HCP-Ph] “When you work with a patient that has got that influence within a community, particularly within an online community, then you can really get that product or that solution out there and you can build that trust because patients trust other patients like themselves.” [P6-PE]	
**Novel approaches**			
	Patient experts (n=13, 65%)	“I think some of the facilitators are now called patient entrepreneurs. We see this a lot in the area of diabetes because they are the experts. And you have to see a GP or specialist very regularly, and then you will quickly realize that you’re the expert and not the physician is the expert.” [P12-DE] “And you see this happening more and more often, where patients are being treated as consultants.” [P8-TP-Ph]	
	Patient engagement agencies (n=4, 20%)	“But now with rising communities and patients’ agencies that are coming up there and so on, I think we’re now at the point where we are only able to get the voice into the complete process.” [P5-PE-DE] “There are now agencies as well that would help us connect in their indications.” [P9-Ph]	
Embedding them in the development teams (n=12, 60%)			“But then I think they should be part of the team or at least of the advisory board so you don’t lose focus of that. It’s also kind of a strategic decision.” [P12-DE] “You would need patients right in there on the innovation teams driving the agenda.” [P13-HCP-TP]
Expectation management (n=7, 35%)			“You need to be very clear in terms of managing their expectations as to the context of what technology can, cannot do, what the aim is and that we’re not going to solve everything here.” [P14-TP-Ph] “And it also helps manage expectations at this stage, to be really transparent and make sure that we understand each other and why, technically, some things are just not feasible, even though we wanted to do them.” [P9-Ph]
Moderation services (n=6, 30%)			“Sometimes part of the investment needs to be in professional facilitators to manage activities and also neutral facilitators are actually going to facilitate a more fair and open engagement process, which will reduce the risk of bias because there is that kind of risk that you might lead patients or your co-creative partners down a certain route that you hope the process is going to go.” [P6-PE] “If you really want to do this systematically, you probably have to work with an external partner who are experts in doing this. And I think that from what I have seen, a lot of startups, they kind of miss that point.” [P12-DE]

### Embedding Patients in All Cycles of the Development Process

To complete the picture, we systematically discussed each stage of the human-centered approach to design and asked each participant to assess the maturity of patient engagement in each of those phases on a scale of 1 to 5, with 1 being the least mature and 5 being the most mature. In addition to the usual 5 design phases, we asked the participants to assess the maturity of patient engagement in life cycle management. We added this step because, given the constant development of these tools, it is crucial to consider life cycle management; otherwise, they become obsolete in a couple of years if the developers do not cope with technological changes and the new tools that come to the market every day.

The early and middle stages of development were assessed as less mature, while later stages, such as prototyping and delivering the solution, were assessed as more mature, meaning that patients tend to be more involved in these stages of development. Patient engagement maturity was assessed as the lowest in life cycle management after the solution was delivered. Table 4 shows the average and SD of patient engagement maturity at each stage of the HCD, as assessed by the participants.

The maturity assessment was well aligned with participants’ views on phases of the design where patients are most or least engaged, and the most common comment was that patients are usually engaged “too late” in the process, at a stage where it is difficult to make any radical changes to the design. The key themes and subthemes, their frequencies, and some sample quotes about phases in which patients are least or most involved in eHealth development are summarized in Table 5 for clarity.

**Table 4:** Maturity assessment of patient engagement in the key phases in human-centered design as assessed by the participants.

Phase of the human-centered design	Maturity assessment (out of 5), mean (SD)
Specify context	2.5 (0.8)
Define user requirements	2.7 (1.2)
Produce design	2.3 (0.9)
Prototype	3.3 (1.0)
Deliver solution	3.7 (0.8)
Life cycle management	2.2 (0.8)

**Table 5:** Phases where patients are least or most involved in eHealth development as expressed by the participants (N=20).

Theme			Sample quotes
**Most involved**			
	At the end (too late; n=14, 70%)	“And in general, the patient engagement happens a little too late in the process.” [P11-PE] “I think it’s at testing, right. That’s really no doubt that’s the only place, which means there is already a prototype. It doesn’t mean it’s already done, but it’s hard to change a prototype, right, at that stage.” [P16-HCP-Ph] “Basically, when the industry needs the patient for dissemination of an app, of support service, then they’ll give it to the patient community, but very often too late.” [P17-PA] “If I take my experience, they were mainly involved at the end. Too late. Way too late.” [P19-PE-DE] “I think the main barrier is actually creating the product first and then looking for feedback second. It needs to be reversed. It needs to be the other way around.” [P6-PE]	
	At the very beginning and very end (n=6, 30%)	“I think some people tend to ask at the beginning, ‘What are your needs?’ And you do 10, 15 interviews at the very beginning. And then you develop what you think it has to be developed. And then, you ask them at the very end.” [P19-PE-DE] “But it’s very often more probably—in the need definition, you have also more engagement, so before the design of the solution and then for the testing.” [P2-PE-DE]	
	At the beginning (n=2, 10%)	“I would say at the beginning.” [P10-DE] “So generally, I think patients tend to be involved in the early phase. When a company is coming up with the design, they kind of have been told, ‘Oh, you need to do patient engagement.’ So, they’ll hold a focus group, they’ll put some post it notes on a wall.” [P15-TP]	
**Least involved**			
	In the middle (n=6, 30%)	“I think they are least involved in the middle phases.” [P19-PE-DE] “this is a massive gap in the middle, where patients aren’t being involved.” [P15-TP]	

**Figure 3:**
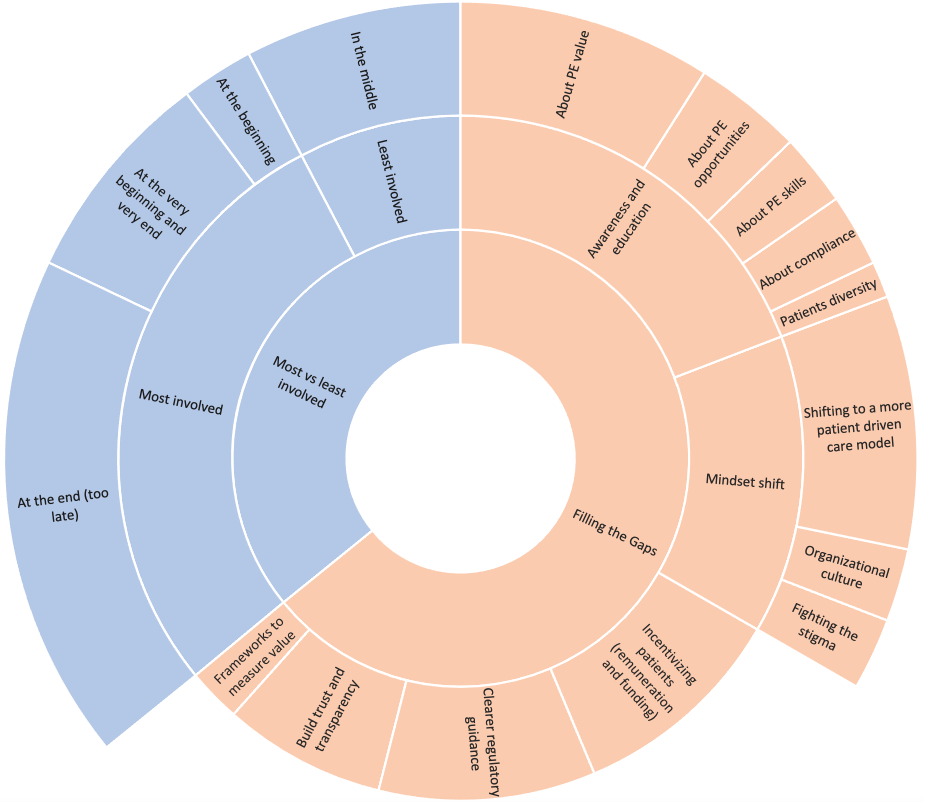
Gaps in patient engagement in eHealth development and how to overcome them. PE: patient engagement.

We further asked them to brainstorm ideas that may help overcome the gaps in the areas where patients are least involved in the development process to ensure that they are embedded as cocreators in all development cycles. Figure 3 shows the themes that emerged as a response to these 2 questions and their respective subthemes, reflecting the frequency of each theme (frequencies reflect the number of participants who mentioned that specific theme).

Participants determined that awareness and education is a key factor that may help overcome the current gaps in patient engagement in eHealth development. This encompasses awareness around the value that patient engagement may bring (7/20, 35%), about available patient engagement opportunities (3/20, 15%), and also about educating patients to equip them with the required skills for efficient patient engagement (3/20, 15%), educating eHealth providers about compliance and the right processes to compliantly engage patients in the development (2/20, 10%), and raising awareness about patient diversity and the differences between different patient profiles and skills (1/20, 5%).

Furthermore, a mindset shift is needed to enable the change. Shifting to a more patient-driven care model would encourage more patient engagement (7/20, 35%), fighting the stigma surrounding being a patient to empower patients to speak up more (2/20, 10%), and an organizational culture change on the solution providers’ side to help embrace patient engagement (2/20, 10%).

Clearer regulatory guidance may also encourage solution providers to engage patients without being too worried about potential regulatory issues (8/20, 40%), incentivizing patients in a compliant manner would encourage patients and providers to partner together in the development process (8/20, 40%), building trust and transparency among the concerned stakeholders could also facilitate the collaboration (6/20, 30%), and finally developing frameworks to measure the value of patient engagement would help build the business case and encourage developers to dedicate the necessary resources for proper patient engagement (2/20, 10%).

The key themes and subthemes, their frequencies, and some sample quotes on how to overcome the gaps in patient engagement in eHealth development are summarized in Table 6 for clarity.

**Table 6:** Participants’ suggestions on how to overcome the gaps in patient engagement in eHealth development (N=20).

Theme		Sample quotes
**Awareness and education**		
	About patient engagement value (n=7, 35%)	“Making sure that people understand and have hands-on experience of what’s the value that patient engagement brings.” [P16-HCP-Ph] “Probably being more aware for the companies about the real benefit for the solution in having patients involved might be something that, let’s say, can influence decision makers and having them participating.” [P10-DE]
	About PE^a^ opportunities (n=3, 15%)	“I think from a patient side, patients have no idea that these opportunities exist. So, you’re not reaching the right patients. They don’t know.” [P7-PE-TP] “Maybe also it’s relevant to create awareness on the developer side how patients can be involved and over which platforms.” [P20-PE-DE]
	About PE skills (n=2, 10%)	“I would argue training to make everybody far more adept at utilizing health research terminology, patient advocacy terms, and sort of the training capacity stuff that EUPATI is doing.” [P11-PE] “I think just getting that insight on what it is and how you do it, those are things which are trainable, right.” [P14-TP-PH]
	About compliance (n=2, 10%)	“Just having a better understanding of the regulatory landscape. I’m not even asking to change the full regulatory landscape. I’m just asking for making it simple enough to be understood, so small companies that don’t have these regulatory teams can actually understand it, and it’s actionable, and they know what to do. And doing a lot more education.” [P19-PE-DE] “I’ve been helping the recruitment of global head of regulatory affairs recently; my role was to assess the patient engagement capacity. And the couple of people I interviewed, none of them were aware. They were delighted to see what I was sharing with them, but they were not aware. They didn’t know what the EMA is doing.” [P17-PA]
	About patients’ diversity (n=1, 5%)	“I think it’s actually promoting the perception, the difference between a lay and a patient expert.” [P11-PE]
**Mindset shift**		
	Shifting to a more patient-driven care (n=7, 35%)	“I have a personal belief that actually the patient community should actually lead these efforts by putting out what are the unmet needs. And not as a response to briefing from one organization with one objective which is already pretty well-defined.” [P17-PA] “But just look at how that consumer journey happens in other industries and perhaps bring lessons learned from those other industries into health care. I think the consumer aspect is really key to look at other ways around how a patient will be engaging and expecting to have their journey improved.” [P4-TP-PH]
	Fighting the stigma (n=2, 10%)	“This was really a time where you had to hide everything of being a chronic patient—people living with a chronic disease… I think it will, hopefully, get less stigma. And patients will be more and more able to speak up.” [P5-PE-DE]
	Organizational culture (n=2, 10%)	“It takes a lot of courage for the organization to say, let’s try out whatever the new tech players offer to not have to build the wheel all of the time and have a fast time to patient time to release on top of that.” [P4-TP-Ph]
Clearer regulatory guidance (n=8, 40%)		“Having more clarity around that. And it’s almost like having clear guidelines within the health system, that this is- the way with clinical trials and so on.” [P13-HCP-TP] “To have some sort of, I don’t even want to call it organization. It can even be a website. But somewhere where the regulatory landscape is easy and that they help you. Because that would help reduce the resistance and the fears inside of the companies.” [P19-PE-DE]
Incentivizing patients (n=8, 40%)		“Create the right level of incentive or engagement, where it’s no longer a volunteer activity, but it is—let’s just use the term, it’s a clear contract with clear ins and outs.” [P14-TP-Ph] “I think it’s important to incentivize people in some way because most people can’t be bothered to give their feedback or—yeah, give a real motivation as to why people should get involved.” [P7-PE-TP]
Build trust and transparency (n=6, 30%)		“It’s this trust factor. So, building trust with patients, spending time with them, to align on how you want to work together.” [P6-PE] “Because participation is also built on trust and so everything has to be very transparent and clear.” [P10-DE]
Frameworks to measure value (n=2, 10%)		“We need to admit that whether you are for or against patient engagement until we measure the value and so on, it’s kind of anti-discussion. Now it’s time to actually measure what works, what doesn’t work, measure the quality.” [P17-PA] “Could somebody quantify whether that actually has a statistical difference in the outcome? That’s very, very hard to do because every tool is so different. But if you could quantify that, then you create a business case for leaders to invest in this direction.” [P8-TP-Ph]

^a^PE: patient engagement.

## Discussion

### Principal Findings

This study shows that genuinely engaging patients in all phases of eHealth development can provide different types of value. The most prominent added value is that engaging patients since the early stages of development would help identify unmet needs, which is crucial because previous research showed that patient needs impact adoption, meaning that a tool that addresses a real need would be more successfully adopted [9,34]. Better usability and desirability are also outcomes of efficient patient engagement in addition to a better fit into the overall patient journey, another central factor for eHealth success, as highlighted by other researchers [35].

Ultimately, patient engagement leads to better eHealth adoption and stickiness, a fact pinpointed by 2 extensive systematic reviews that concluded that involving users in the development leads to better adoption [35,36]. Sustained adoption and tool stickiness may eventually lead to better health outcomes, as pointed out by other studies that highlighted the positive link between patient activation and treatment compliance [37] and showed an enhancement in patients’ health outcomes and better quality of care with sustainable eHealth use [38,39]. In addition, considering that the digital divide is mostly considered a barrier to adoption [35,40], the inclusion of diverse profiles of patients with different skillsets and capabilities may contribute to the creation of more inclusive designs that lead to better equity and access to health care.

However, despite the invaluable contributions that patients may bring, the study participants agreed that they are usually engaged too late in the development process, mostly assuming a sounding role to test a ready-made prototype, as opposed to being embedded as equal partners and cocreators throughout the different phases of development. This aligns with the findings of a previous systematic review of international experiences that also determined that patient involvement is mostly achieved through consultation and that direct participation is less common [41].

This low engagement may be explained by some prominent barriers, notably compliance. The participants highlighted regulatory processes’ complexity and lack of clarity as critical obstacles that hinder patient engagement. It was also noted that many stakeholders’ perception of exaggerated regulatory risks creates resistance and reluctance to efficiently engage patients in the development. The challenge of compliance aligns with the findings of other researchers who pointed out negative attitudes toward engaging patients, especially concerning patient safety, as one of the key barriers to patient involvement [7,10].

The study participants also cited patient-related barriers, most prominently challenges in patient identification and access, primarily due to regulatory processes. In addition, factors such as health constraints depending on the patient’s condition, as pointed out by previous research showing that a patient’s health condition may impact how they engage with health care technologies [42] and sometimes lack the necessary skills for efficient engagement, were also recognized as potential hurdles.

Moreover, the power dynamics in the health care sector are generally not in patients’ advantage, resulting in a general perception that they are more of passive receivers of care than equal partners in their health management. The economic model puts the power in the payers’ hands, which are typically not the patients themselves but rather insurance companies; similarly, the decision power mostly lies in the clinicians’ hands rather than the patients. This imbalance of power, paired with potential conflicts of interest among the key stakeholders, disfavors patients and results in situations where they find themselves not given a safe space to actively and equally contribute to discussions impacting their own health.

Other barriers include engaging patients in a “check-in-the-box” activity. Undertaken to look good on paper, without real essence, which is typically due to the lack of understanding of the value that genuine patient engagement may bring, as mentioned by other researchers, stressing that the lack of standardized best practices and metrics has made it challenging to achieve consistency and measure success in patient engagement [43]. Furthermore, embedding patients in the development process is a resource-intensive undertaking that requires time and budget that may not always be available. Besides mistrust issues, mainly driven by health data management concerns around eHealth tools, and the lack of transparency of some stakeholders, making it harder to gain patients’ trust, an issue that has also been emphasized by other researchers [44,45].

On the positive side, the participants reflected on some facilitators that may enable better patient engagement. For instance, working with engagement partners such as patient organizations may help overcome some of the regulatory hurdles. Clinicians can also play an active role in engagement, as noted by other researchers [46]. Engaging through the care team can help developers access not only patients who are already actively participating in patient organizations and advocacy groups but also most patients who are least active but may bring an essential perspective that would otherwise be missing. Active involvement in patient communities, offline and web-based, may also facilitate the collaboration between patients and their caregivers. Novel approaches, such as the rise of professional patient experts and patient engagement agencies, are also furthering the collaboration between the different stakeholders by simplifying the matchmaking process and helping overcome some patient identification and access barriers.

Practices that facilitate patient engagement include embedding the patients in the development teams, meaning hiring them as project managers or user experience experts if they have the necessary skill set. Suppose the involved patients do not necessarily have professional expertise and know-how to understand technical discussions. In that case, hiring professional moderation services that can help translate the language between patients and the development team and active expectation management, explaining what is possible or not possible, and what is in scope or not, can play a significant role in enabling successful collaboration between all parties.

### Blueprint for Patient Engagement as Cocreators of eHealth Technologies

On the basis of the understanding of the value of patient engagement in eHealth development, its current state of maturity, and potential barriers and facilitators, we propose an end-to-end practical blueprint that can guide the relevant stakeholders to successfully engage patients as equal partners and cocreators in all phases of the HCD rather than mere testers of preplanned prototypes.

The first layer of the blueprint addresses sample considerations and the specific patient profiles that may best suit each phase of the HCD. Bearing in mind that the middle phases of the design are the least mature from a patient engagement perspective, partly because of the technical skills required to contribute to these phases efficiently, we suggest engaging with professional patient experts in the 3 middle phases. Working with patient experts ensures that the involved parties are equipped with the disease experience and the necessary know-how to engage in meaningful development discussions. The newly rising patient engagement agencies may help overcome patient identification and access barriers by matching the development teams with suitable patient experts. Organizations such as the European Patients’ Academy on Therapeutic Innovation (EUPATI) also help match patient experts with health care researchers through their EUPATIConnect services [47].

However, it is crucial to warrant the diversity of the patients, especially in the first and last phases of the design, and to avoid working solely with patient experts in all phases. Involving lay patients in the mix and people who are not necessarily technically savvy will help the development team to create an inclusive design that is still usable even for the least capable users, enabling more health care equity and reducing the unbalancing effect of the digital divide. There are clinics and hospitals that have started establishing innovation laboratories, such as the University Hospital in Basel Switzerland [48], enabling the testing and cocreation of new health technologies with clinicians and lay patients who volunteered to test these tools.

The middle layer of the blueprint presents recommendations for the most suitable engagement approach for each phase. During the first phase of the design, the development team focused on specifying the context, including evidence review. Potential engagement approaches during this early phase may vary from monitoring discussions in web-based patient communities, looking directly into patient complaints or requests in clinics and hospitals, and conducting patient workshops or focus groups. One-to-one interviews may also provide in-depth insights, primarily when conducted in the patients’ natural environment, to best reflect the entire patient journey and unaddressed needs.

As soon as the second phase of the design begins and the development team starts defining user requirements, the discussion becomes more technical. This is when professional moderation services of workshops and focus groups gain importance, as they can enhance the chances of a mutual understanding between stakeholders with varying technical skills. Ideation sessions using a design-thinking approach and benchmarking of existing apps are crucial tools at this stage.

The development teams sometimes merge the third and fourth phases of the design, producing the design and prototyping. However, it is worth noting that it may be worth starting with some A/B testing when producing the design, a way to compare 2 versions of a single variable, typically by testing a subject’s response to variant A against variant B and determining which of the 2 variants is more effective [49]. For example, this approach may be used to test language and design elements before moving to prototyping. This saves time and effort by ensuring that the basic design resonates with the patients before producing the prototype. Simulations and laboratory and in-field testing are very relevant at this stage, as they help developers better understand actual user behavior rather than solely relying on self-reported feedback through surveys or checklists.

When delivering the tool, it is vital to test it in a real-life setting to ensure its fit into the patients’ journey, meaning that it fits well into their daily routines and wholistic treatment plans. Beta-testing or piloting can be valuable in allowing developers to test their tool in a real-life setting on a smaller scale before rolling it out. It is also advised to have a hypercare period immediately after the launch of any eHealth tool, where developers closely monitor user analytics and platform metrics to act swiftly in case of any issues, providing a smoother integration in a real-world setting.

The bottom layer of the blueprint addresses the often-neglected life cycle management, which was assessed as the least mature from a patient engagement perspective. Ensuring an iterative approach that actively manages the constant development of eHealth tools is critical for sustainable success, especially in an ever-changing technical landscape. Continually engaging with patients through consistent life cycle management ensures the stickiness and relevance of the tool. Engagement approaches can be as simple as actively monitoring and responding to the support line and email, or app-store feedback, but can also be more proactive, such as periodically engaging patient key opinion leaders to obtain their input. Other useful tools in this stage are drip email systems to constantly seek users’ feedback and transparent communications about new iterations to inform users how their feedback was taken into account in the constant development of the tool.

**Figure 4:**
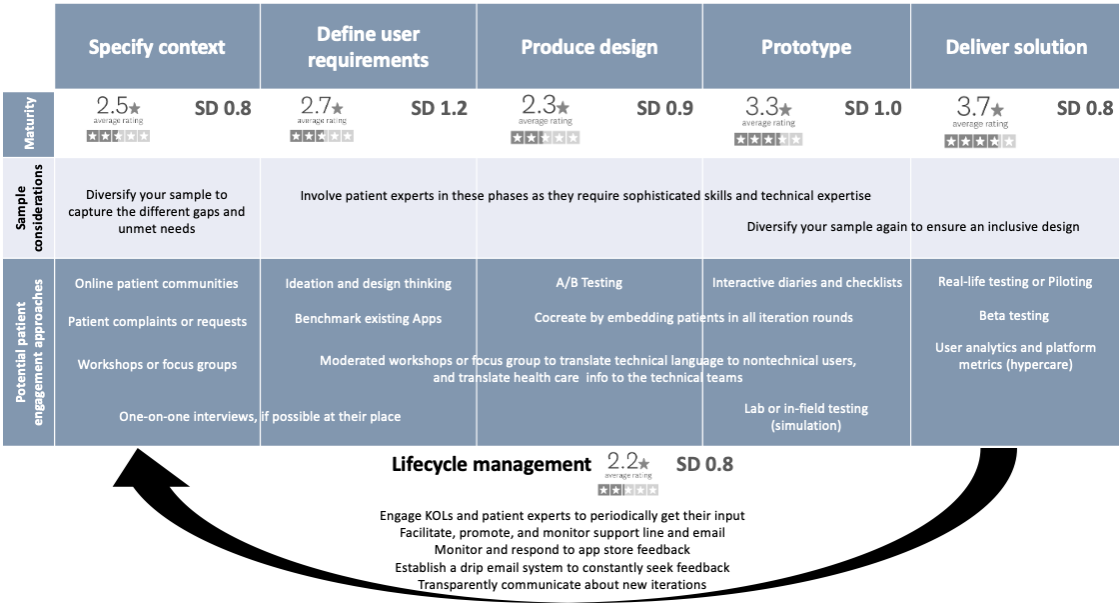
Proposed blueprint for patient engagement as cocreators of eHealth technologies. KoL: key opinion leaders.

Figure 4 shows the proposed blueprint for patient engagement in every phase of the HCD, presenting suggestions for patient sample considerations and recommendations for the most suitable engagement approaches for each phase.

### Practical Implications

Overcoming the current gaps in patient engagement in eHealth development requires consolidated efforts from all stakeholders in the complex health care ecosystem. Policy makers, clinicians, eHealth providers, pharmaceutical companies, insurance companies, patient organizations, advocacy groups, and health care innovation incubators must work hand in hand to induce change and harness the potential value that true cocreation with patients can bring.

Education and awareness are key to improving patient engagement. On the one hand, it involves educating patients and equipping them with the necessary knowledge and skills for effective engagement and contribution. Organizations such as EUPATI are already actively providing patient education programs [50]; however, more efforts are needed in the area of eHealth and all that it entails from specific technical skills. On the other hand, it is crucial to raise awareness of the value that patient engagement can bring, provide platforms that may help promote patient engagement opportunities, and provide more information about relevant compliance processes.

The study participants emphasized that there is a need for measurement frameworks that can help quantify the impact of patient engagement, as similarly highlighted in the systematic review by Bombard et al [51] and stressed by other scholars [52]. Some initiatives are digging deeper into this issue in attempts to create tools that may help evaluate patient engagement; for example, the Public and Patient Engagement Evaluation Tool developed at McMaster University in Canada [53,54] and its Norwegian expansion Evalueringsverktøy for Brukermedvirkning [55]. Furthermore, citizen-led organizations such as Patient-Focused Medicines Development and PARADIGM, a public-private partnership coled by the European Patients’ Forum and The European Federation of Pharmaceutical Industries and Associations, are working on metrics that aim to help better monitor and evaluate patient engagement [56,57]. These efforts can help shed light on the business case for patient engagement to overcome the value perception barrier and enable genuine patient engagement as equal partners and cocreators.

Regulatory clarity and simplification would play a central role in facilitating patient engagement, given that compliance was deemed the most prominent barrier by study participants. A clear step-by-step approach, templates for agreements and contracts, clarity on patient remuneration, and health data privacy and management would encourage the relevant stakeholders to engage patients compliantly without being too worried about compliance risks. Owing to this lack of globally accepted guiding principles around patient involvement that identify and integrate good practices, organizations such as Patient-Focused Medicines Development work with patients and other stakeholders to cocreate frameworks and toolboxes that may guide the relevant stakeholders in their patient engagement efforts. Their patient engagement synapse provides sample agreements and contracts to facilitate compliant engagement and collaboration between patients and providers of medical technologies [58]. Another example of the type of guidance required is the Workgroup of European Cancer Patient Advocacy Networks Guiding Principles on Reasonable Legal Agreements between patient advocates and pharmaceutical companies [59]. This could also help solve the patient remuneration and incentivization dilemma as it offers clear guidance on the type of contract that may enable the collaboration in a compliant manner.

Barriers such as patient access and identification could be overcome by working with engagement partners and promoting novel approaches to empower professional patient experts and innovators with visibility and networks. The rise of matchmaking services can also play a positive role in overcoming these barriers; patient engagement agencies that focus on identifying and engaging with relevant patient experts to match them with suitable patient engagement opportunities are a good example. There are also organizations such as EUPATI that offer matchmaking services through their EUPATIConnect, as mentioned earlier [47]. Equally, we can overcome potential mistrust between different stakeholders with complete transparency and disclosure of collaborations and partnerships, as well as more clarity on data management practices.

This change would also require a mindset shift on several levels. First, a shift toward more value-based health care would help overcome the current imbalance in power dynamics and reinforce a more active role for patients; research has shown that only once patients are allowed to participate in managing their health actively, they take ownership of their disease management, thus improving health outcomes [60]. Second, it is key to fight the stigma around disease and being a patient, to empower patients, and to encourage them to speak up. Third, overcoming risk aversion toward patient engagement in health care organizations is a significant factor closely linked to regulatory clarity and simplification, as well as awareness of the value that genuine patient engagement may bring.

**Figure 5:**
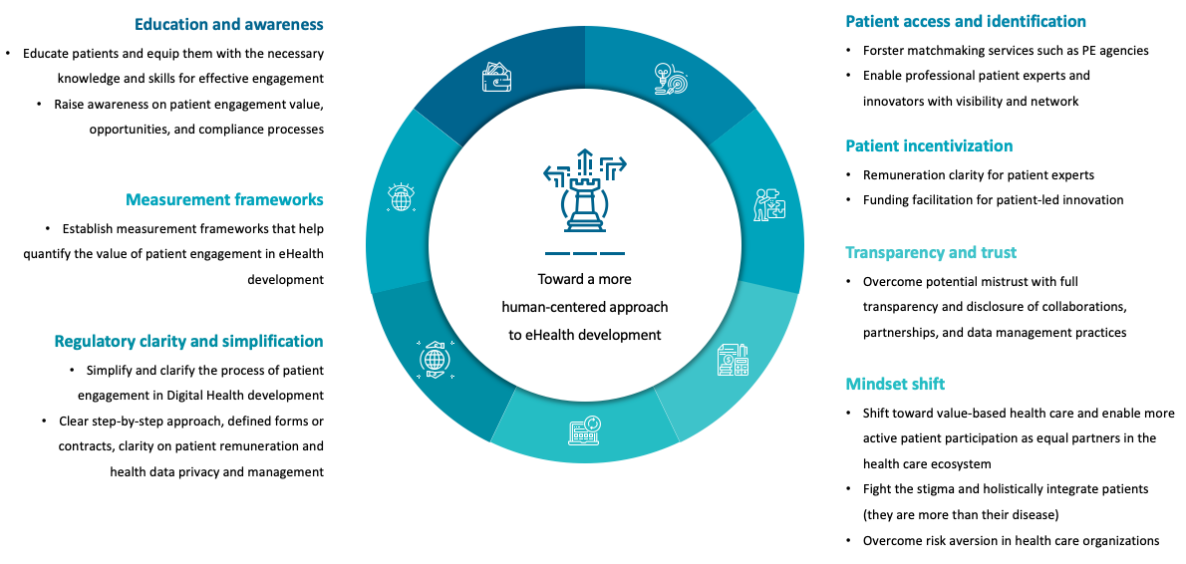
Recommendations for more patient-driven eHealth development. PE: patient engagement.

Figure 5 shows the key practical implications of this study and our recommendations for more patient-driven eHealth development.

### Limitations and Recommendations for Future Research

This qualitative study has some limitations that we would like to outline. Our study was limited to 6 countries in a specific timeframe, and generalization to other settings that might have different characteristics, such as a different regulatory landscape, may be challenging. Moreover, the relatively small sample size and dynamic nature of eHealth necessitate a constant update of the findings to cope with the changes. Future research may address some of the cited limitations by covering other countries, timeframes, regulatory frameworks, and settings.

### Conclusions

The outcome of this study contributes to creating awareness about the value of genuine patient engagement, barriers, and facilitators that impact engagement efforts and how to overcome the current gaps. We propose a blueprint that considers these specific findings and aims to facilitate the successful engagement of patients as cocreators in all phases of HCD rather than mere testers of preplanned prototypes.

Our findings highlight the tremendous value created by patient engagement in eHealth development. However, it also emphasizes the dominant gaps in the current patient involvement approaches. We shed light on the compelling obstacles behind these gaps and discuss ways to overcome them. It is important to note that overcoming the current gaps in patient engagement in eHealth development requires consolidated efforts from all stakeholders in the complex health care ecosystem, not only relying on medical technology providers to overcome them, as some factors go beyond their direct control.

## Appendix

Multimedia Appendix 1 Interview guide.

Multimedia Appendix 2 Survey Monkey form.

Multimedia Appendix 3 Phases of thematic analysis after Braun and Clarke [28,29].

Multimedia Appendix 4 Barriers to patient engagement as expressed by the participants.

## Abbreviations

EUPATI: European Patients’ Academy on Therapeutic Innovation
HCD: human-centered design
