# Supplementary material for: From Testers to Cocreators—the Value of and Approaches to Successful Patient Engagement in the Development of eHealth Solutions: Qualitative Expert Interview Study
Source: JMIR Hum Factors. 2022 Oct 6;9(4):e41481. doi: 10.2196/41481 (PMC9585443; doi:10.2196/41481)
Supplement: Multimedia Appendix 1 [file humanfactors_v9i4e41481_app1.pdf]

Interview Guide

From testers to co-creators: the value and approaches to successful patient engagement in the development of digital health solutions

Introduction

Hello. Thank you for agreeing to talk to me. My name is (…) and I am working on a research project on behalf of PersonalPulse to explore the topic of better patient engagement in DH development.

The aim of this study is to create a research-based end-to-end practical blueprint that can guide the relevant stakeholders to successfully engage patients as co-creators in all human-centered design phases rather than mere testers of preplanned prototypes.

I will not ask you to discuss your current employer or to provide any confidential or proprietary information. Instead, you are invited to talk about opportunities or specific challenges and best practices within your scope of expertise.

There are no right or wrong answers and if you have any worries or concerns then just stop and ask me.

With your consent, the discussion will be recorded for the sake of the transcription and analysis.

Participant's Background

1. Please tell me a bit about yourself

- What is your experience with Digital Health?
- How have you been involved in patient engagement in the past?

Setting the Stage

2. What comes to your mind when you think of patient engagement?

3. To ground the rest of our discussion, please tell us in a nutshell about 1-2 different digital health solutions you worked on.

- Please describe the solution, its goals, end users, & how patients were engaged in the development process

Barriers and Facilitators

4. In your experience, how does patient engagement bring value to DH development? *e.g. different ways they contribute to the value and success, unmet needs, better understanding of patient journey, better chances for adoption*

5. Tell me about some of the barriers you faced or you have witnessed to the involvement of people who are patients in DH developments? *e.g. different types Barriers (patient related, developer related, resource related…)*

6. And what are the facilitators? opportunities?

Filling the gaps

7. In your experience, what are the areas of DH design where patients are most involved? What roles do they typically play?

8. What are the areas they are least involved in? Why do you think that is? *i.e. specific barriers?*

9. What can be novel approaches to patient involvement in the co-creation to overcome the current gaps, and what are their success factors (i.e., must haves for such approaches/modalities to work)? *e.g. patient selection and experience*

The HCD blueprint for PE

Work with the participants to systematically identify the level of Patient Engagement (PE) maturity and activities at each stage of the Human-centered design (HCD)

10. Let's structure the input you gave us so far into the different phases of Human-centered design to ensure a systematic patient engagement in all waves of the design process. We will look into the PE maturity at each wave, and the different PE activities that may be performed

**Phase**	**Inspiration**		**Ideation**	**Implementation**	
**Wave**	**Specify Context** *Evidence Review*	**Define user requirements** *User Research*	**Produce Design** *Concept testing*	**Prototype** *Evaluate against requirements*	**Deliver Solution** *Usability testing*
**PE maturity** *Likert scale 1-5 (1 least mature, 5 most well established)*					
**Current and potential PE activities**					

11. How do you ensure constant input from patients even after delivering the solution? *Mechanisms for ongoing engagement*

Closing

12 . Is there anything you would like to add?

- Is there any question you expected us to ask but we didn't?

Thank you very much for your valuable contributions
