# Supplementary material for: From Testers to Cocreators—the Value of and Approaches to Successful Patient Engagement in the Development of eHealth Solutions: Qualitative Expert Interview Study
Source: JMIR Hum Factors. 2022 Oct 6;9(4):e41481. doi: 10.2196/41481 (PMC9585443; doi:10.2196/41481)
Supplement: Multimedia Appendix 2 [file humanfactors_v9i4e41481_app2.pdf]

From testers to co-creators: the value and approaches to successful patient engagement in the development of digital health solutions

Work with the participants to systematically identify the level of Patient Engagement (PE) maturity and activities at each stage of the Human-centered design (HCD)

* 1. Participant initials

2. Specify Context (*Evidence Review*)

Least mature ☆ ☆ ☆ ☆ ☆ Most well established | N/A ○

3. Define user requirements (*User Research*)

Least mature ☆ ☆ ☆ ☆ ☆ Most well established | N/A ○

4. Produce Design (*Concept testing*)

Least mature ☆ ☆ ☆ ☆ ☆ Most well established | N/A ○

5. Prototype (*Evaluate against requirements*)

Least mature ☆ ☆ ☆ ☆ ☆ Most well established | N/A ○

6. Deliver Solution (*Usability testing*)

Least mature ☆ ☆ ☆ ☆ ☆ Most well established | N/A ○

7. Lifecycle management (*mechanisms for ongoing engagement after launch*)

Least mature ☆ ☆ ☆ ☆ ☆ Most well established | N/A ○

Done

Powered by
SurveyMonkey
See how easy it is to create a survey.

Privacy & Cookie Notice
