# Supplementary material for: From Testers to Cocreators—the Value of and Approaches to Successful Patient Engagement in the Development of eHealth Solutions: Qualitative Expert Interview Study
Source: JMIR Hum Factors. 2022 Oct 6;9(4):e41481. doi: 10.2196/41481 (PMC9585443; doi:10.2196/41481)
Supplement: Multimedia Appendix 4 [file humanfactors_v9i4e41481_app4.pdf]

Multimedia Appendix 4. Barriers to patient engagement as expressed by the participants.

Theme	Sample quotes
**Compliance**	
**Complexity (n=18**, 90 %**)**	
	• "Even people that want to make it happen, then they face massive compliance, regulatory, practical, legal issues that actually you need to be a kind of super warrior to engage patients in most organizations today." [P17-PA]<br>• "there's a lot of question marks about how you engage with patients and how you get their feedback in a compliant manner." [P3-TP]
**Patient compensation (n=9**, 45 %**)**	
	• "The key barrier is also payment. Sort of not remunerating patients on an equal footing for their contribution and time" [P11-PE]<br>• "I guess the financial side of it, I guess that falls into regulatory. Obviously, you'd be happy to compensate them for their time, but how would you do that in a compliant manner?" [P3-TP]
**Perception (n=9**, 45 %**)**	
	• "There are a lot of gray areas. And there is this fear of, 'Oh my God, if we do something in these gray areas and then something happens, the consequences are devastating'." [P19-PE-DE]<br>• "For some of the players, it starts with, unfortunately, the mentality which was developed, regulations are there, and they're perfectly fine. We need guard rails, but it drove, unfortunately, this mentality that if you're within a research organization or pharma, you actually never get to speak or talk to a patient." [T4-TP-Ph]

	**Process clarity (n=7**, 35 %)	
		• "It wasn't that people didn't want to engage patients. It was that people just have no idea how to do it." [P19-PE-DE]<br>• "And another one is actually a lack of process and know-how to engage with patients." [P6-PE]
**Patient related**		
	**Patient identification (n=13**, 65 %**)**	
		• "the identification of the patients or the kind of the persona and the underlying demographics play actually a crucial role. E.g., differing age results in differing ability to use digital solutions." [P20-PE-DE]<br>• "It's about finding the right patients. It's not just about—so often I see it's like, 'Oh, we've involved patients,' and they've interviewed two patients or even 10 patients or whatever. It doesn't matter. You need people that truly understand the problem." [P7-PE-TP]
	**Patient access (n=11**, 55 %**)**	
		• "Other aspect of it is some actually don't know where to even find the patients. Where do we find these patients? And some are not aware that there's a huge amount of, again, different types of patients. It depends on if there are patients that have got a level of expertise that can really add something extra." [P6-PE]<br>• "There is an access issue, I think. So, a lot of companies, although they claim to be experts in the disease area, actually aren't embedded in the patient community. So, they're having to go through maybe doctors or charities to gain access to those patients. And it adds like a wall

		for them that they don't have that direct conversation." [P15-TP]
	**Health constraints (n=4**, 20 %**)**	
		• "In some cases, we had problems in having them involved, for example, in workshops, because due to some impairments they had in moving, there are some constraints to participation." [P10-DE]<br>• "Some patients will not want to roll their sleeves. Maybe they're just too tired." [P16-HCP-Ph]
	**Lack of skills (n=1**, 5 %**)**	
		• "Information and communication technology skills can be a barrier in the interaction since some people are unfamiliar with the usage of it." [P20-PE-DE]
**Power dynamics**		
	**Patients not seen as equal partners (n=9**, 45 %**)**	
		• "I think it's this feeling that it's going to be so complicated and so complex, and that patients don't really understand the process. So, their input is not going to be valid." [P19-PE-DE]<br>• "And a lot of patients don't feel like they have something to give. Healthcare is very much this, 'We're going to fix you. You're just a patient. What do you know?'" [P7-PE-TP]
	**Conflict of interests (n=7**, 35 %**)**	
		• "often there is a mismatch of expectations of what a patient wants from what's actually economically pragmatic." [P11-PE]<br>• "Maybe there's also a difference in terms of the motivation. I think in healthcare when patient and physician, I think there is a common motivation to promote the healthcare, to improve quality of life. But if there is, let's say, an external provider like developers, they may

		have other motivation; let's say extrinsic, so monetary aspects." [P20-PE-DE]
	**Patients not given a safe space (n=4**, 20 %**)**	
		• "Patients can sometimes feel scared or overwhelmed to speak up if they don't agree with something, particularly if it's doctors in the room, or a Pharma company, or they feel bamboozled by some of the language." [P15-TP]<br>• "In our kind of culture... the doctor is going to fix you. They know everything. You don't question them. So, you don't feel like you're going to be listened to or valued." [P7-PE-TP]
	**Economic model (n=4**, 20 %**)**	
		• "The main problem is that they are not involved that much because they are not an economic player in the system. So, the economic power, but also the power to really decide." [P10-DE]<br>• "But given where the economic power balance rests, patient input will always be the icing on the cake, the nice to have. Maybe gives a more positive experience. But from a purely commercial and a business perspective, does it create a higher revenue stream?" [P14-TP-Ph]
	**Decision power (n=3**, 15 %**)**	
		• "The decision is normally taken by whatever heads of departments and administrative people and physicians. But they are not the users, but they are the ones who decide." [P18-HCP-DE]<br>• "But I think it's not the patients who get asked the question. It's probably the people paying the money." [P8-TP-Ph]
	**PE as lip service, marketing, and CSR[a] (n=12**, %**)**	
		• "I think this is also—if the motivation is not honest, maybe sometimes this is also like

	ticking a box. Okay. Patients involved, check. Let’s go to the next phase.” [P12-DE]<br><br>• “I’ve come across some companies who are genuinely, totally convinced that they’ve worked with patients to co-create—but in reality, all they’ve done is to just show the finished product to a group of patients for their seal of approval.” [P6-PE]
**Mistrust (n=11**, 55 %**)**	
	• “There’s a mistrust of working in health and certain segments of communities because they’ve had largely negative experiences.” [P11-PE]<br><br>• “And in the terms of trust, I think also to mention that data protection is really important.” [P20-PE-DE]<br><br>• “it’s not always super easy, at least when you come from industry. Because the moment they see you come from industry, it’s, ‘Oh, another one that wants something from us.’ I think there is a perception that we are going to use them.” [P19-PE-DE]
**Resources (n=11**, 55 %**)**	
	• “And then obviously having the resources, it all takes time. As you know, if you want to do it properly, you have to really spend a lot of time with us to do the transcripts of the focus groups, the interviews, whatever, and then come back, iterate and go back again. There’s a huge amount of time and effort and resources can go into it.” [P13-HCP-TP]<br><br>• “But sometimes, it’s always the trivial thing. Very often, it’s money.” [P18-HCP-TH]
**Value perception (n=11**, 55 %**)**	

	• "I think it comes back to some of the things I was saying around barriers, where lack of understanding around the value that they could bring, just seeing them as a patient, not as someone with life experience. The patient that you're interviewing might turn out to be a lead web developer at some place, or they have pertinent experience to your product." [P15-TP]<br>• "The patient engagement or advocacy functions are at a mercy (budget wise) of people who may not have that full understanding of the value of patient engagement." [P16-HCP-Ph]
**Inflexibility (n=4, %)**	
	• "And sometimes people don't want to be told that what they're developing isn't the right thing." [P15-TP]<br>• "There are founders out there, they are hiding the product for years because they are afraid to share it and to get real feedback." [P5-PE-DE]<br>• "It's also important to maintain flexibility. So don't be surprised if the process takes you in a direction that you weren't expecting and to be ready for that and to be willing to learn from that." [P6-PE]

aCSR: corporate social responsibility.
